# Induction of Oxidative Stress in SH-SY5Y Cells by Overexpression of hTau40 and Its Mitigation by Redox-Active Nanoparticles

**DOI:** 10.3390/ijms24010359

**Published:** 2022-12-26

**Authors:** Natalia Pieńkowska, Margaret Fahnestock, Crystal Mahadeo, Izabela Zaborniak, Paweł Chmielarz, Grzegorz Bartosz, Izabela Sadowska-Bartosz

**Affiliations:** 1 Laboratory of Analytical Biochemistry, Institute of Food Technology and Nutrition, College of Natural Sciences, Rzeszow University, 4 Zelwerowicza Street, 35-601 Rzeszow, Poland; 2 Department of Psychiatry and Behavioural Neurosciences, McMaster University, 1280 Main Street West, HSC-4N80, Hamilton, ON L8S 4K1, Canada; 3 Department of Physical Chemistry, Faculty of Chemistry, Rzeszow University of Technology, Al. Powstańców Warszawy 6, 35-959 Rzeszow, Poland; 4 Department of Bioenergetics, Food Analysis and Microbiology, Institute of Food Technology and Nutrition, College of Natural Sciences, Rzeszow University, 4 Zelwerowicza Street, 35-601 Rzeszow, Poland

**Keywords:** tau protein, SH-SY5Y cells, oxidative stress, reactive oxygen species, glutathione, nitroxide, nitroxide-containing nanoparticles

## Abstract

Abnormally phosphorylated tau protein is the principal component of neurofibrillary tangles, accumulating in the brain in many neurodegenerative diseases, including Alzheimer’s disease. The aim of this study was to examine whether overexpression of tau protein leads to changes in the redox status of human neuroblastoma SH-SY5Y cells. The level of reactive oxygen species (ROS) was elevated in tau-overexpressing cells (TAU cells) as compared with cells transfected with the empty vector (EP cells). The level of glutathione was increased in TAU cells, apparently due to overproduction as an adaptation to oxidative stress. The TAU cells had elevated mitochondrial mass. They were more sensitive to 6-hydroxydopamine, delphinidin, 4-amino-TEMPO, and nitroxide-containing nanoparticles (NPs) compared to EP controls. These results indicate that overexpression of the tau protein imposes oxidative stress on the cells. The nitroxide 4-amino-TEMPO and nitroxide-containing nanoparticles (NPs) mitigated oxidative stress in TAU cells, decreasing the level of ROS. Nitroxide-containing nanoparticles lowered the level of lipid peroxidation in both TAU and EP cells, suggesting that nitroxides and NPs may mitigate tau-protein-induced oxidative stress.

## 1. Introduction

In human brain studies and experimental models of Alzheimer’s disease (AD), oxidative stress has been shown to play a critical role in neurodegeneration [1,2]. Currently, there is no effective treatment for AD. It should be noted that the principal pathological features of AD include plaque deposition composed of β-amyloid (Aβ) peptide as well as deposition of neurofibrillary tangles composed of the abnormally hyperphosphorylated tau protein [3]. Liu et al. reported that tau accumulation is pathologically more relevant to the development of neurodegeneration and cognitive decline in AD patients than Aβ plaques [4].

Alzheimer’s disease and other neurodegenerative diseases (such as progressive supranuclear palsy [5], frontotemporal dementia [6], and corticobasal degeneration [7]), where the accumulation of pathological tau protein in the brain occurs, are referred to as “tauopathies”. Even though the phenotypic manifestation of specific cell types and brain areas affected vary among tauopathies, the presence of accumulated tau protein in the absence of other pathological hallmarks in tauopathies substantiates the toxicity of tau, independent of Aβ [8].

Tau protein belongs to the family of microtubule-associated proteins (MAPs) and in the healthy brain is responsible for microtubule stabilization and plasticity, with the ability to influence axonal growth, transport, and neuronal polarization, and hence the normal function of neurons [6,9]. It has more than 40 identified phosphorylation sites [10]. In the diseased brain, tau becomes abnormally hyperphosphorylated, causing microtubule destabilization and affecting axonal transport. Free-floating soluble tau protein then begins to aggregate until insoluble tau aggregates are finally sequestered as intracellularly neurofibrillary tangle (NFT) lesions [9]. It is important to note that in pathological conditions, the accumulation of insoluble tau aggregates can occur inside neurons as well as in the extracellular space and in other brain cells such as astrocytes and oligodendrocytes [11].

Disturbances in the structure and/or expression of proteins, especially those prone to aggregation, may be another source of oxidative stress in neurons. Overexpression or misfolding of alpha-synuclein protein is associated with increased ROS production. It was reported that overexpression of alpha-synuclein, especially its mutant forms, increases intracellular ROS levels and exaggerates the vulnerability of neurons to dopamine-induced cell death [12]. Oxidative stress, in turn, may contribute to alpha-synuclein aggregation [13], thus completing a vicious circle perpetuating oxidative stress. There are many clues suggesting that changes in the structure, phosphorylation, and expression of the tau protein may have similar consequences. Overexpression of wild-type tau in N2a neuroblastoma cells resulted in increased susceptibility to hydrogen peroxide, linked to peroxisome depletion in neurites due to inhibition of transport along microtubules [14]. Neurons from transgenic rats expressing a human truncated tau protein analogous to a variant form derived from sporadic AD showed higher sensitivity to agents inducing oxidative stress, such as glucose oxidase and buthionine sulfoximine (inhibitor of GSH synthesis) [15]. Tau protein hyperphosphorylation has been proposed to induce and perpetuate oxidative stress [16], although the exact mechanism of induction of oxidative stress in this case remains elusive.

The aim of this study was to examine whether overexpression of the tau protein leads to changes in the redox status of SH-SY5Y human neuroblastoma cells and induces oxidative stress. Moreover, the effects of both natural (genistein and delphinidin) and synthetic antioxidants, a nitroxide (4-amino-TEMPO, 4-AT), and nitroxide-containing redox nanoparticles (NPs) were examined. Delphinidin was found to interfere with the mechanisms of tau protein aggregation [17], inhibit tau hyperphosphorylation [18], and inhibit spatial memory impairment and AD hallmarks in Meynert lesioned rats in an animal model of AD [19]. Genistein has been reported to inhibit dopaminergic neuronal death [20] and was suggested to have potential for the delay and treatment of AD [21,22]. Redox nanoparticles containing nitroxide residues have been found to be beneficial in cellular models of neurodegenerative diseases [23,24] and to inhibit amyloid plaque propagation in the Tg2576 mouse model of AD [25]. Our previous studies have demonstrated beneficial effects of free nitroxides and, especially, NPs in a cellular model of Parkinson’s disease (SH-SY5Y cells treated with 6-hydroxydopamine (6-OHDA)) [26,27].

## 2. Results

**Figure 1:**
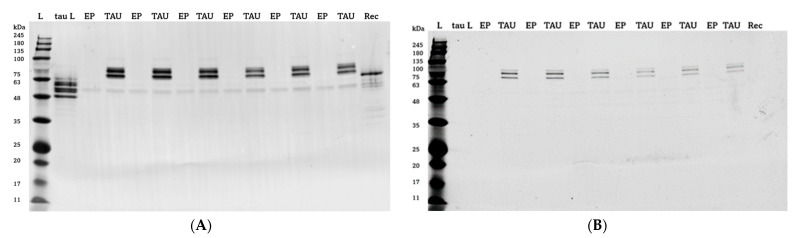
hTau40 was overexpressed in non-differentiated SH-SY5Y cells (**A**) and was also hyperphosphorylated (**B**). Blots of 12% gels; proteins visualized by anti-total Tau antibody (**A**) and anti-phospho-Tau (Thr 181) antibody (**B**), respectively. (**A**): Lanes: 1, protein ladder; 2, human tau ladder (tauL), 2 μg; 3, 5, 7, 9, 11 and 13, EP cell lysates, 5 μg/well; 4, 6, 8, 10, 12 and 14, TAU cell lysates, 5 μg/well; 15, recombinant Tau 441 protein (Rec), 0.1 μg. **B**: all lanes are the same as in (**A**).

Non-differentiated SH-SY5Y cells were stably transfected with a plasmid carrying the sequence of hTau40 (the longest human tau isoform). hTau40 overexpression was confirmed via Western blotting (Figure 1A,B). Plasmid-derived tau protein has a V5 tag that adds approximately 4 kDa to the protein.

**Figure 2:**
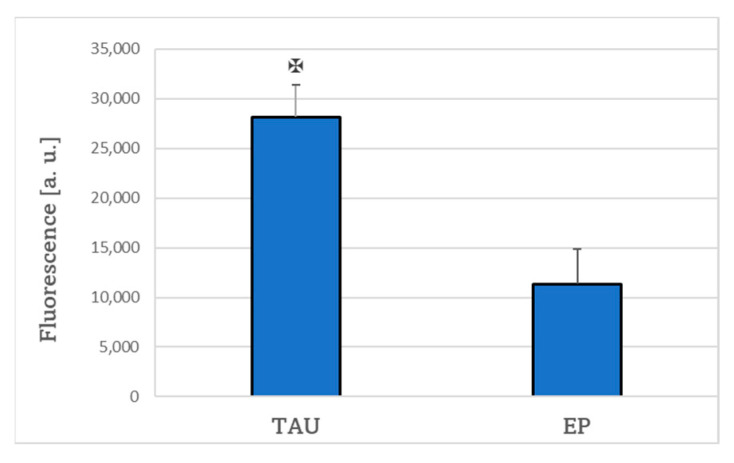
Fluorescence of protein aggregates in TAU-transfected cells was significantly higher compared to empty plasmid- transfected cells (EP), as measured by Aggresome staining; ✠ *p* < 0.05 (TAU vs. EP); *n* = 3.

The level of protein aggregates was higher in cells transfected with the Tau isoform (TAU) cells than in cells transfected with the empty plasmid (EP cells) (Figure 2).

**Figure 3:**
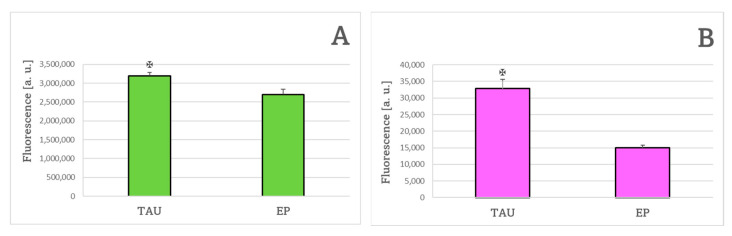
ROS level in TAU cells was significantly higher compared to EP controls as estimated with 2′,7′-dichlorodihydrofluorescein diacetate (**A**) and dihydroethidium (**B**) after 24-h treatment. ✠ *p* < 0.05 (TAU vs. EP); *n* = 3.

The level of reactive oxygen species (ROS) was elevated (by 15 ± 6% when estimated by H_2_DCF-DA and by 119 ± 19% when estimated with DHE) in TAU cells as compared to the EP controls (Figure 3). 

**Figure 4:**
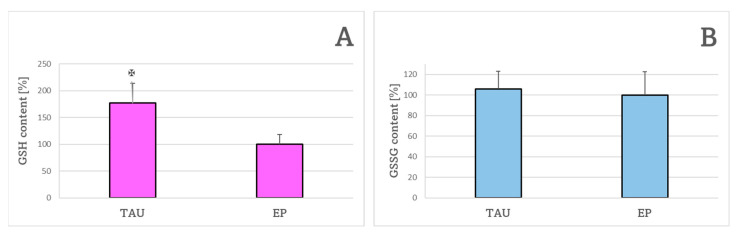
The content of reduced glutathione (**A**) and oxidized glutathione (**B**) in TAU and EP SH-SY5Y cells after 24 h. GSH and GSSG content in EP cells assumed as 100%. ✠ *p* < 0.05 (TAU vs. EP); *n* = 3.

The content of reduced glutathione (GSH) was significantly elevated in TAU cells compared to EP cells (Figure 4A). The content of oxidized glutathione (GSSG) was not significantly altered in TAU cells compared to EP cells (Figure 4B).

**Figure 5:**
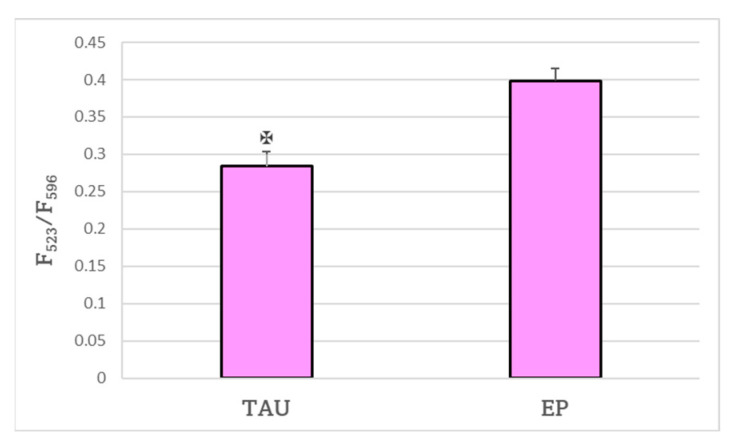
TAU cells had a significantly lower level of lipid peroxidation compared to EP control cells as estimated with the BODIPY C11 probe. The ratio of fluorescence measured at 523 and 596 nm after 24 h was used as a measure of lipid peroxidation. ✠ *p* < 0.05; *n* = 3.

The level of lipid peroxidation estimated with the BODIPY C11 probe was considerably lower in TAU cells compared to EP cells (Figure 5).

**Figure 6:**
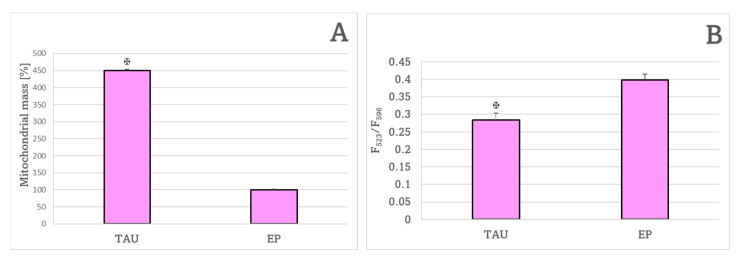
Mitochondrial mass (**A**) and red/green fluorescence ratio of the JC-1 probe, a measure of mitochondrial membrane potential (**B**) after 24-h treatment. (**A**) TAU cells had significantly higher mitochondrial mass compared to EP controls. The mitochondrial mass value obtained for EP cells was assumed as 100%. (**B**) TAU cells showed a significant decrease in the red/green fluorescence ratio of the JC-1 probe compared to EP controls. ✠ *p* < 0.05 with respect to non-treated cells; *n* = 3.

The mitochondrial mass was significantly increased in TAU cells with respect to EP cells (Figure 6A). The red to green fluorescence ratio, a measure of the mitochondrial inner membrane potential, was significantly lower in TAU cells than in EP cells (Figure 6B).

**Figure 7:**
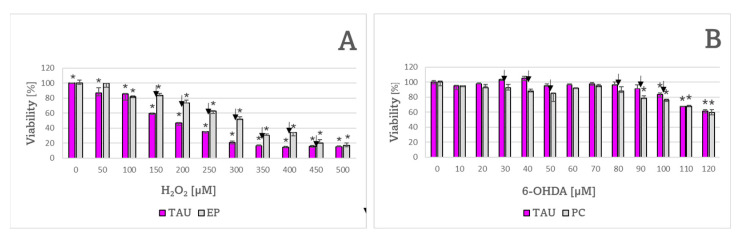
Sensitivity of TAU and EP cells to H_2_O_2_ and 6-OHDA. TAU cells were significantly more sensitive to H_2_O_2_ at concentrations ranging from 150 to 450 μM (**A**) and more resistant to 6-OHDA at concentrations of 30 to 50, and 80 to 100 μM compared to EP controls (**B**). * *p* < 0.05 with respect to cells not treated with H_2_O_2_ or 6-OHDA. ^↓^ *p* < 0.05, TAU compared to EP (cells treated with the same concentration of H_2_O_2_ or 6-OHDA); *n* = 3.

Sensitivity of TAU and EP cells to several compounds affecting the cellular redox equilibrium, both oxidants and antioxidants, was measured and compared. TAU cells were more sensitive to 150–450 μM hydrogen peroxide compared to EP cells (Figure 7A) and more resistant to 30 μM, 40 μM, 50 μM, and 80–100 μM 6-OHDA than EP cells (Figure 7B).

**Figure 8:**
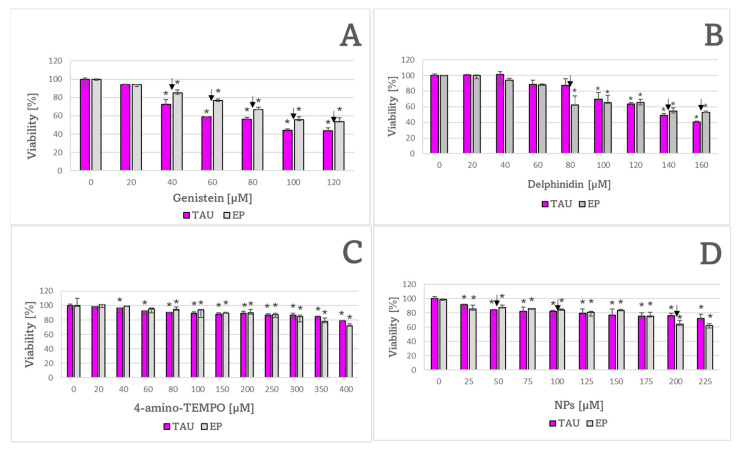
Sensitivity of TAU and EP cells to genistein (**A**), delphinidin (**B**), 4-amino-TEMPO (**C**), and NPs (**D**). * *p* < 0.05, compared to no treatment controls; ^↓ ^*p* < 0.05, TAU cells vs. EP cells treated with the same concentration of the antioxidant; *n* = 3.

TAU cells were more sensitive to 40–120 μM genistein (Figure 8A) but more resistant to 80 μM delphinidin (Figure 8B) than EP cells. Additionally, TAU cells were more resistant to 200 μM NPs compared to EP controls (Figure 8D).

On the basis of IC_50_ values of the compounds tested, it can be concluded that TAU cells were more sensitive to hydrogen peroxide and genistein than EP cells (Table 1).

**Figure 9:**
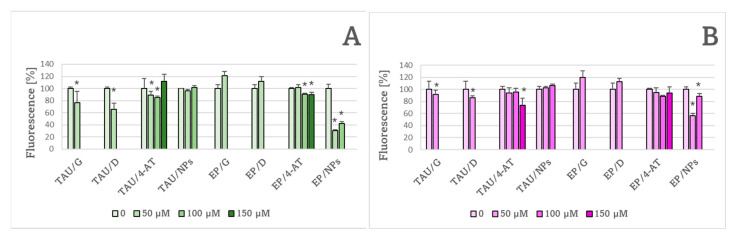
Effect of 24 h treatment with 50 μM genistein (G) and delphinidin (D) and of various concentrations of 4-amino-TEMPO (4-AT) and nitroxide-containing redox nanoparticles (NPs) on ROS levels in TAU and EP cells, estimated with 2′,7′-dichlorodihydrofluorescein diacetate (**A**) and dihydroethidium (**B**). * *p* < 0.05, compared to cells not treated with the antioxidant; *n* = 3.

The antioxidants ameliorated oxidative stress and its consequences in SH-SY5Y cells. Both H_2_DCF-DA (Figure 9A) and dihydroethidium (Figure 9B) detected diminution of ROS levels by genistein, delphinidin, and 4-AT in TAU cells and by NPs in EP cells. The results obtained with H_2_DCF-DA point also to diminution in the ROS level in EP cells by 4-AT (Figure 9A). 

**Figure 10:**
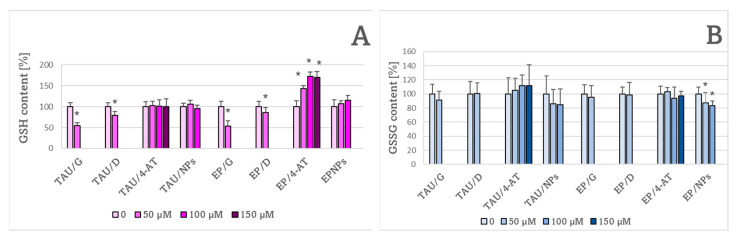
Effect of 50 μM genistein (G) and delphinidin (D) and of various concentrations of 4-amino-TEMPO (4-AT) and nitroxide-containing redox nanoparticles (NPs) on GSH (**A**) and GSSG (**B**) content in TAU and EP cells after 24-h treatment. * *p* < 0.05 with compared to cells not treated with an antioxidant; *n* = 3.

Treatment with the natural antioxidants genistein and delphinidin caused a decrease in the GSH content in both TAU and EP cells. 4-AT did not change the GSH content in TAU cells but caused an increase in the GSH content in EP cells, while NPs did not affect the GSH content in either cell type (Figure 10A). It should be recalled that the GSH level was significantly augmented in TAU cells with respect to EP cells (Figure 4). Only NPs significantly affected the GSSG content, decreasing it in EP cells (Figure 10B).

**Figure 11:**
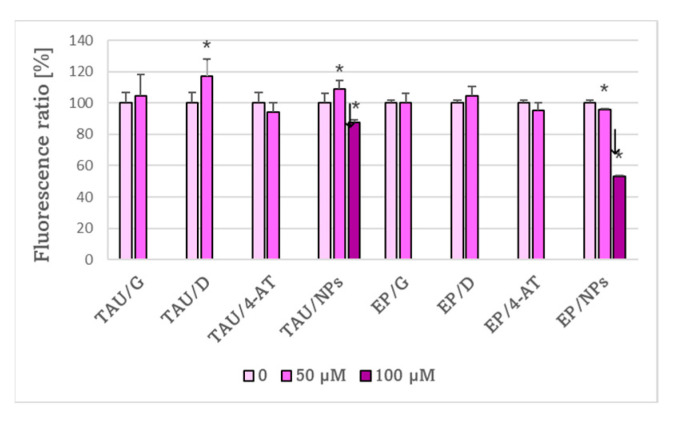
Effect of antioxidants on the level of lipid peroxidation in TAU and EP cells. G, genistein; D, delphinidin; 4-AT, 4-amino-TEMPO; NPs, nanoparticles. * *p* < 0.05 compared to cells not treated with the antioxidant; ^↓ ^*p* < 0.05, TAU cells compared to EP cells treated with the same concentration of antioxidant; *n* = 3.

Delphinidin significantlyenhanced while 100 μM NPs significantly decreased lipid peroxidation probed by BODIPY 11 in TAU cells. Nitroxide-containing nanoparticles also decreased lipid peroxidation in EP cells (Figure 11). It should be noted that the level of lipid peroxidation was higher in EP than in TAU cells (Figure 5).

**Figure 12:**
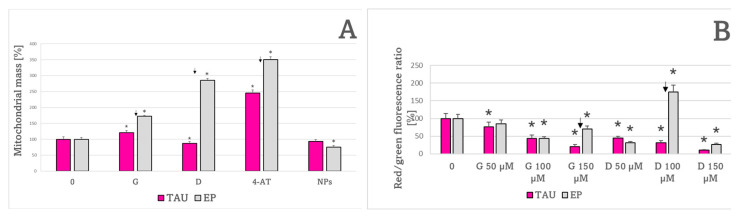

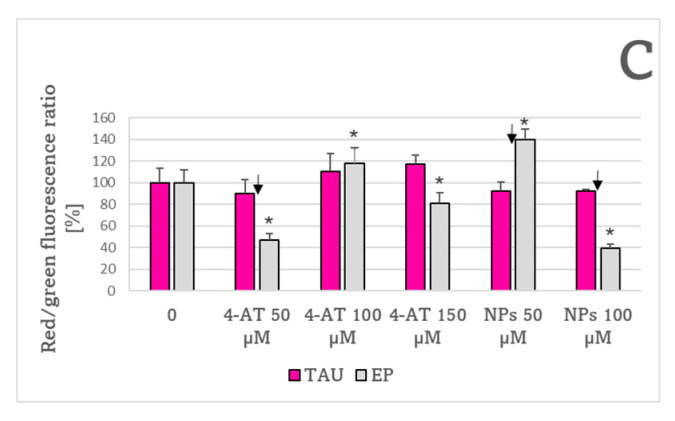
Effect of antioxidants (50 μM) on mitochondrial mass (**A**) and concentration dependence of the effects of antioxidants on the JC-1 red to green fluorescence ratio (**B**,**C**) after 24 h as a measure of the mitochondrial membrane potential in TAU and EP cells. Values of the mitochondrial mass and JC-1 fluorescence ratio are expressed as percent of values in cells of respective lines not subjected to any treatment (“0”). G, genistein; D, delphinidin. * *p* < 0.05, compared to cells not treated with the antioxidant; ^↓^ *p* < 0.05, TAU cells compared to EP cells treated with the same concentration of the antioxidant; *n* = 3.

Treatment with genistein increased mitochondrial mass in TAU and EP cells compared to untreated cells (Figure 12A). Delphinidin increased mitochondrial mass in EP cells. 4-AT increased the mitochondrial mass in both cell types. The increase was lower in the case of TAU cells compared to EP cells, but the mitochondrial mass was dramatically increased in TAU cells in the absence of any treatment (Figure 6). Redox nanoparticles decreased the mitochondrial mass in EP cells.

The effects of antioxidants on the mitochondrial potential of SH-SY5Y cells werestrongly concentration- and cell-type-dependent. Changes in JC-1 red/green fluorescence ratio point to changes in the mitochondrial potential. The concentration of 100 μM delphinidin increased the mitochondrial potential in EP cells; other concentrations of genistein and delphinidin, except for 50 μM genistein which had no significant effect on EP cells, decreased the mitochondrial potential in both TAU and EP cells, with respect to the untreated cells (Figure 12B). The 50 μM and 150 μM 4-AT decreased while 100 μM 4-AT increased the mitochondrial potential in EP cells. NPs increased the mitochondrial potential at the concentration of 50 μM and decreased it at a concentration of 100 μM and had no significant effect on the mitochondrial potential in TAU cells (Figure 12C).

## 3. Discussion

In the present study, non-differentiated SH-SY5Y cells were used. It can be argued that differentiated cells are a worse model of postmitotic neurons than non-differentiated cells. However, it was reported that differentiated SH-SY5Y cells exhibit alterations in the Akt pathway, resulting in higher tolerance to 6-OHDA toxicity. Furthermore, in addition to inducing cell differentiation, retinoic acid (RA) can trigger survival signaling in different cell types. As a result, RA-differentiated cells are less susceptible to Parkinsonism mimetic than undifferentiated cells, and so undifferentiated SH-SY5Y may be more appropriate for studying neurotoxicity or neuroprotection in experimental Parkinson’s disease research [28,29].

The present results demonstrate the occurrence of oxidative stress in tau-overexpressing SH-SY5Y cells as evidenced by an elevated level of ROS and enhanced GSH content compared to empty-vector controls. An increased level of protein aggregates in TAU cells compared to EP cells suggests that excess tau protein is involved in protein aggregate formation. The enhancement of the GSH content represents most probably an adaptive response to oxidative stress as was previously demonstrated for oxidative stress induced by 6-OHDA in SH-SY5Y cells [26,30], a model of Parkinson’s disease [31,32,33,34,35,36,37,38,39,40,41,42,43]. No significant changes in the content of GSSG in TAU cells suggest efficient regeneration of GSH by the glutathione reductase system. A significant increase in the mitochondrial mass in TAU cells compared to EP cells was also found. The enhanced mass of mitochondria in TAU cells may be responsible for enhanced generation of ROS, as mitochondria are the major source of ROS in neurons [34,35]. An inverse relationship may also hold. Lee et al. suggested that the increase in mitochondrial mass in replicative senescent cells may result from an increase in ROS production, and that it is dependent on both de novo synthesis of nuclear-DNA-encoded proteins and their import into mitochondria [36]. It was reported that an increase in mitochondrial mass and mtDNA are the molecular event6s associated with increased oxidative stress in human cells with impaired respiratory function caused by mtDNA deletion [37].

Interestingly, TAU cells were more sensitive to hydrogen peroxide and genistein than control cells, as evidenced by lowered IC_50_ values, which may be a consequence of pre-existing oxidative stress. 

Various hypotheses have attempted to explain how disturbances in the structure and function of the tau protein result in neurodegenerative diseases, postulating both loss and gain of function [38]. The possible involvement of oxidative stress in the underlying mechanism of changes to tau structure and function in the development of tauopathies is intriguing. It has been demonstrated that oxidative stress may be a common mechanism of action of various cell-death-inducing agents, independent of the primary mechanism of their action [39]. It is tempting to speculate that a similar situation can affect neuronal cells in neurodegenerative disorders. There is abundant evidence of oxidative stress in neurodegenerative disorders, although the question of whether oxidative stress is the cause or effect of these disorders is still open [34,40,41,42,43]. Various mechanisms of induction of oxidative stress in neurodegenerative diseases have been proposed, including augmented ROS production in the mitochondria of neurons and glia, reactions of transition metal ions, reactions of dopaquinones, increased activity of monoamine oxidase, activation of NADPH oxidase, activation of N-methyl-D-aspartate receptor, and inflammation [34,44,45,46]. Consequences of oxidative stress in the central nervous system include such key features of neurodegenerative diseases as accumulation of protein aggregates, increase in intracellular free Ca^2+^, release of excitatory amino acids, autophagy, loss of trophic support and apoptosis. All these mechanisms play a critical role in the course of many neurological disorders including Parkinson’s and Alzheimer’s diseases and amyotrophic lateral sclerosis [47].

The present data demonstrate that overexpression of hTau40 alters the redox equilibrium and induces oxidative stress in SH-SY5Y cells. These results support other data suggesting the role of oxidative stress in tauopathies leading to neurodegeneration. Using the *Drosophila* model, oxidative stress was demonstrated to mediate neuronal cell death in transgenic flies that express a disease-related mutant form of human tau (tauR406W) in a panneuronal pattern [48]. Other studies confirmed that oxidative stress is a causal factor in tau-induced neurodegeneration in *Drosophila* [49]. These results suggested that increased levels of oxidative stress play an active role in enhancing tau-mediated neurodegeneration, possibly through cell cycle activation, underscoring the therapeutic potential of targeting antioxidant pathways and cell cycle mechanisms for the treatment of AD and other human tauopathies [48]. What is more, a fragment of tau protein has been shown to induce copper reduction, thus contributing to oxidative stress and initiating copper-mediated generation of H_2_O_2_ [50]. Krishnamurthy et al. suggested that oxidative-stress induced cell death occurs through both caspase-dependent and -independent pathways, and that tau is likely an in situ substrate of caspase-3 [51]. Interestingly, it was demonstrated that chronic oxidative stress induced in vitro via buthionine sulfoximine inhibition of glutathione synthesis increases the levels of tau phosphorylation at paired helical filament epitopes (serine 396/404) [52].

Modulation of the endogenous antioxidant barrier and application of exogenous antioxidants affected the cytotoxic effects of tauR406W expression [48]. Genetic modulation of antioxidant defense cannot be considered in human neurodegenerative diseases; nevertheless, modulation of oxidative stress by antioxidants is a fully available mode of ameliorating these diseases [53,54,55]. In the present study, the effects of both natural (genistein and delphinidin) and synthetic (a nitroxide, 4-AT and NPs) antioxidants were examined. Two fluorogenic probes, H_2_DCF-DA and DHE, were used in this study. While DHE is more specific for superoxide, H_2_DCF-DA is not selective in its reactions, being oxidized by all ROS formed in a cell [56]. Both H_2_DCF-DA (Figure 9A) and DHE (Figure 9B) detected diminution of ROS levels by genistein, delphinidin and 4-AT in TAU cells and by NPs in EP cells. The results obtained with H_2_DCF-DA point also to the diminution of ROS levels in EP cells by 4-AT (Figure 9A).

Nitroxides such as 4-AT are synthetic stable free radicals stabilized by methyl groups at the α position in six-membered piperidine or five-membered pyrrolidine, pyrroline, or oxazolidine ring structures. The methyl groups confer stability to the nitroxide radicals by preventing radical–radical dismutation and limiting access to reactive substances, which can quench other free radicals [57]. Nitroxides have antioxidant properties, which include a pseudoenzymatic superoxide dismutase activity, reactivity with various free radicals, and inhibition of Fenton reaction and lipid peroxidation, as well as protection of proteins from glycoxidation, nitration, and oxidation [58,59,60,61]. 4-Amino-TEMPO and NPs decreased the level of ROS detectable with H_2_DCF-DA in TAU or EP cells, respectively. Genistein and delphinidin decreased the level of GSH in TAU and EP cells, while 4-AT increased in in EP cells. 4-AT increased the mitochondrial mass in TAU cells. Genistein and delphinidin decreased the mitochondrial potential in TAU cells, which does not seem to be a beneficial effect, while 4-AT had no significant effect. Higher concentrations of the antioxidants were cytotoxic themselves, and the results obtained are biased to some extent by this effect in the case of genistein and 100 μM delphinidin. However, 4-AT and NPs induced beneficial modifications of some oxidative stress-related parameters while having no deleterious effect on other parameters.

The amelioration of oxidative stress by 4-AT and NPs suggests that they are good candidates for in vivo applications, first in animal models of neurodegenerative diseases. Nitroxides and NPs are able to penetrate the blood–brain barrier [26], which increases their chances to be effective in vivo. Attaining in vivo concentrations so high as to exhibit cytotoxicity does not seem probable.

Nitroxide-containing redox-nanoparticles can be more effective than low-molecular weight nitroxides, especially in vivo, since their elimination from the body is much slower and, if taken up, they reside inside the cells. Nitroxide-containing nanoparticles show good antioxidative properties in vitro and were considered as potential treatments against various diseases [24,62,63]. Their effectiveness in amelioration of oxidative stress in tau-overexpressing SH-SY5Y cells observed in this study suggests that they may be promising candidates to mitigate neuronal oxidative stress and delay the progress of various tauopathies.

## 4. Materials and Methods

### 4.1. Materials and Equipment

The human neuroblastoma cell line originating from neural tissue [SH-SY5Y (CRL-2266)] was obtained from the American Type Culture Collection. This cell line is derived from a metastatic bone tumor of a 4-year-old cancer patient. SH-SY5Y cells were stably transfected with the longest human 4-repeat tau isoform, hTau40, subcloned into the pcDNA3.2/V5/DEST vector, as well as with the empty vector (pcDNA3.2) as a control. Both transfection groups were cultured under selective pressure with 300 μg/mL of G418 [64].

Dulbecco’s modified Eagle’s medium/Nutrient Mixture F-12 without phenol red (cat. no. 21041025), Dulbecco’s phosphate-buffered saline (DPBS) (cat. no. 14040-117), and Lipid Peroxidation Sensor (4,4-difluoro-5-(4-phenyl-1,3-butadienyl)-4-bora-3a,4a-diaza -s-indacene-3-un-decanoic, C11-BODIPY^®^ 581/591 (cat. no. D3861)) were purchased from Thermo Fisher Scientific (Waltham, MA, USA). Fetal bovine serum (cat. no. S1813), penicillin–streptomycin solution (cat. no. L0022), Trypsin–EDTA solution (10×) (cat. no. X0930), and phosphate-buffered saline without Ca^2+^ and Mg^2+^ (cat. No. P0750) were obtained from Biowest (Nuaillé, France). G418 disulfate salt (cat. No. A1720), 2-propanol (cat. No. I9516), Thiazolyl Blue Tetrazolium Bromide (MTT) (cat. No. M2128), 2′,7′-dichlorodihydrofluorescein (H_2_DCF-DA) (cat. No. 35845), dihydroethidium (DHE) (cat. No. 37291), 0.4% Trypan Blue solution (cat. No. T8154), 4-amino-TEMPO (cat. No. 163945), *N*-ethylmaleimide (NEM) (cat. No. E3876), trichloroacetic acid (TCA) (cat. No. T4885), diethylenetriaminepentaacetic acid (DTPA) (cat. No. D1133), L-ascorbic acid (cat. No. A0278), dimethyl sulfoxide (DMSO) (cat. No. D2438), *ortho*-phtaldialdehyde (OPA) (cat. No. P1378), N-nonyl acridine orange (NAO) (cat. No. A7847), 6-hydroxydaopamine hydrobromide (cat. No. 162957), Triton X-100 (cat. No. X-100), and Natriumdithionit (cat. no. 15795-3) were provided by Merck (Poznan, Poland). 96% ethanol (cat. no. 396420113) as well as methanol (cat. no. 6219900110) were obtained from Avantor Performance Materials (Gliwice, Poland). Delphinidin chloride (cat. no. 528-53-0) was purchased from EXTRASYNTESE (Genay, France). Genistein (cat. no. sc-3515) was obtained from Santa Cruz Biotechnology, Inc. (Santa Cruz, CA, USA). JC-1 Mitochondrial Membrane Potential Assay Kit (cat. no. AB113850) was obtained from Abcam (Cambridge, UK). Hydrochloric acid (cat. no. 115752837), Folin–Ciocalteu’s reagent (cat. no. 116943507) and hydrogen peroxide 30% (cat. no. 118851931), Edetate Disodium Dihydrate pure (EDTA) (cat. no. 118798103), and Formaldehyde Solution Pure (cat. no. 114321734) were obtained from Chempur (Piekary Śląskie, Poland). EDTA-free inhibitor tablets and PhosSTOP Phosphatase Inhibitor Cocktail Tablets were from Roche (Basel, Switzerland). PROTEOSTAT^®^ Aggresome Detection Kit (cat. no. ENZ-51035) was purchased from Enzo Life Sciences (Lausen, Switzerland). Intercept^®^ Blocking Buffer TBS was from LI-COR Biosciences (Lincoln, NE, USA). Anti-total Tau (Tau 12 antibody, was from Biolegend (San Diego, CA, USA) and anti-phospho-Tau (Threonine 181) were from Cell Signaling Technology (Danvers, MA, USA), and secondary antibodies IRDye^®^ 680 and IRDye^®^ 800CW were from LI-COR Biosciences.

**Figure 13:**
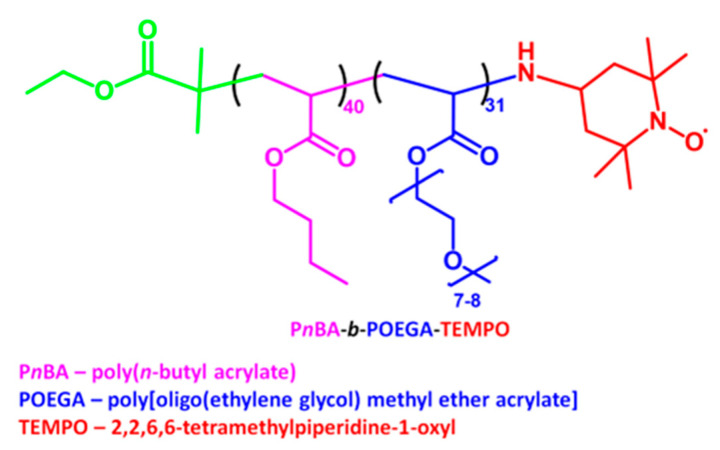
Structure of nitroxide-containing nanoparticles (NPs).

Cell culture 75 cm^2^ flasks (cat. no. 156499), 25 cm^2^ flasks (cat. no. 156340), and the Lab-Tek™ II Chamber Slide™ System (cat. no. 154534) were provided by Thermo Fisher Scientific (Waltham, MA, USA). Transparent 96-well Advanced TCTM culture plates (cat. no 655980), black 96-well flat bottom μClear^®^ Advanced TCTM plates (cat. no. 655986), transparent 96-well (cat. no. 655101), black 96-well flat bottom plate (cat.no. 655209), and 24-well cell culture transparent plates (cat. no. 662160) were obtained from Greiner Bio-One (Kremsmünster, Austria). Other sterile cell culture materials were provided by Nerbe (Winsen, Germany) or Greiner Bio-One (Kremsmünster, Austria). Amphiphilic linear nitroxide-containing nanoparticles (NPs; Figure 13) were prepared by co-polymerization of two types of acrylate monomers, hydrophobic and hydrophilic. Mean molecular weight of the NPs was 20,592; mean hydrodynamic Z-average, determined by dynamic light scattering, was 31 ± 1 nm ([65], manuscript in preparation).

Stock solutions of delphinidin chloride and genistein were freshly prepared in DMSO and diluted in cell culture medium. 4-Amino-TEMPO and MTT were dissolved in PBS, filtered through a 0.22 µm filter before each experiment, and diluted in cell medium. 6-Hydroxydopamine hydrobromide was freshly prepared, stabilized with 0.01% L-ascorbic acid, and filtered using a 0.22 μm syringe filter for each experiment. Distilled water was purified using a Milli-Q system (Millipore, Bedford, MA, USA). Fluorometric and absorptiometric measurements were conducted in a Tecan Infinite 200 PRO multimode reader or a Spark multimode microplate reader (Tecan Group Ltd., Männedorf, Switzerland). Transmission light microscope observations were conducted in an inverted Olympus CKX53 microscope (OLYMPUS, Tokyo, Japan).

### 4.2. Cell Culture Propagation

Cells stably transfected with hTau40 and pcDNA3.2 were grown in DMEM/F12 medium without phenol red supplemented with 1% *v*/*v* penicillin and streptomycin solution and 10% heat-inactivated fetal bovine serum (FBS), additionally enriched with G418 (300 μg/mL). Cells were incubated at 37 °C under 5% carbon dioxide and 95% humidity. Cells were split every 4 days at a ratio of 2:3. Cell morphology was examined under an inverted microscope with phase contrast Zeiss Primo Vert (Oberkochen, Germany). Cell viability was estimated by the trypan blue exclusion test. Cells were counted in a Thoma hemocytometer (Superior Marienfeld, Lauda-Königshofen, Germany).

### 4.3. Protein Extraction

Cells were plated (500,000 cells per well) and then incubated overnight before harvest. Medium was removed and wells were washed once using 1 mL of PBS per well. Cells were then lysed using 150 µL/2 wells (to increase protein concentration) of the lysis buffer on ice for 10 min, scraped, and collected. Cell lysis buffer preparation: 50 mM Tris-HCl, pH 7.4; 150 mM NaCl; 5 mM EDTA; 1% Triton; 1 EDTA-free tablet; and 1 PhosSTOP Phosphatase Inhibitor Cocktail Tablet/10 mL of the lysis buffer and then mixed gently for 20 min. Samples were collected in 1.5 mL tubes and centrifuged for 20 min at 11,290× *g* at 4 °C (Hermle Z 230MR, Labnet International Inc., Cary, NC, USA). The supernatant and pellet were then separated, and both were stored in a −80 °C freezer. A detergent compatible assay (Bio-Rad, Hercules, CA, USA) was used to determine protein concentrations per sample using a BSA standard.

### 4.4. Western Blotting

We used 12% sodium dodecyl sulfate (SDS) polyacrylamide gels to separate 5 µg of total protein from hTau40- and pcDNA3.2-transfected SH-SY5Y differentiated cells before transferring them onto a PVDF (polyvinylidene fluoride) membrane. A tau protein ladder (rPeptide, Watkinsville, GA USA) containing all 6 human isoforms of tau and a recombinant tau 441 (hTau40) protein (rPeptide) were also loaded on the gel as controls. PVDF membranes were blocked for 1 h using Intercept^®^ Blocking Buffer TBS (LI-COR Biosciences) and probed overnight at 4 °C with anti-phospho-Tau (Threonine 181) and anti-total Tau (Tau 12) antibodies at a 1:1000 dilution overnight at 4 °C. Membranes were then incubated with secondary antibody (either IRDye^®^ 680 or IRDye^®^ 800CW) at a 1:10,000 dilution for 1 h at room temperature and scanned using the Odyssey Infrared Imaging System (LI-COR Biosciences).

### 4.5. Staining of Protein Aggregates

To determine the presence of protein aggregates in transfected neuroblastoma cells, a PROTEOSTAT^®^ Aggresome Detection Kit was used. Cells were seeded into an 8-well chamber slide (Lab-Tek™ II Chamber Slide™ System) at a density of 300,000 cells/well and allowed to attach for 24 h. A positive control was prepared by treating SH-SH5Y cells with 10 μM MG-132 (included in the set) for 12 h. Subsequently, cells were carefully washed twice with 150 μL PBS and fixed with 4% formaldehyde for 30 min (200 μL/well). After the second rinse, PBS was removed, and 150 μL/well permeabilizing solution (0.5% Triton X-100, 3 mM EDTA) was added. Chamber slides were placed on ice for 30 min and gently shaken. Cells were washed 2× with PBS, and 200 μL of Dual Detection Reagent was added (1 μL of PROTEOSTAT^®^ Aggresome Detection Reagent and 2 μL of Hoechst 33342 Nuclear Stain per 2 mL of the assay buffer). Samples were protected from light and incubated at room temperature for 30 min. After incubation, the cells were washed, and the coverslip was placed on a microscope slide. The stained cells were analyzed in a fluorescence microscope (Olympus CKX53).

### 4.6. Hydrogen Peroxide and 6-Hydroxydopamine Cytotoxicity

TAU- and EP-transfected cells were seeded into a 96-well clear Advanced TM plate at a density of 4 × 10^4^ cells/well in 100 µL culture medium and allowed to attach for 24 h at 37 °C. Afterwards, the cells were treated for 24 h with hydrogen peroxide at a concentration range of 50–500 µM and with 6-OHDA at a concentration range of 10–100 µM. Stock and working solutions of H_2_O_2_ were prepared in cell medium. A stock solution of 6-OHDA was prepared in PBS, stabilized with 0.01% L-ascorbic acid, filtered using a 0.22 μm syringe filter, and diluted in cell culture medium to a working concentration. After 24 h, the incubation medium was removed and replaced with 100 μL of 0.5 mg/mL MTT solution in PBS and incubated for 2 h at 37 °C. After incubation, 100 µL/well of acidic isopropanol (250:1 isopropanol:HCl) was added, and the contents of the well were thoroughly mixed. The plate was shaken for 20 min (700 rpm) at room temperature. Absorbance was measured at 570 nm.

### 4.7. Cytotoxicity of Antioxidants

TAU and EP cells were seeded into a 96-well clear Advanced TM plate at a density of 4 × 10^4^ cells/well in 100 µL culture medium and allowed to attach for 24 h at 37 °C. Subsequently, cells were treated with genistein, delphinidin, or 4-AT at a concentration range of 10–100 µM and NPs at a concentration range of 25–150 µM. Stock solutions of tested substances were prepared in DMSO or PBS. Working solutions of studied factors were prepared in culture medium. The DMSO concentration was adjusted to 0.2% in all samples, which had no significant effect on treated cell lines. Cytotoxicity of the tested agents was determined by the MTT test as described above.

### 4.8. Level of Reactive Oxygen Species

The level of ROS in TAU and EP cells, untreated and treated with selected antioxidants, was assayed using 2′,7′-dichlorodihydrofluorescein (H_2_DCF-DA) and dihydroethidium (DHE). The cells were seeded in a 96-well flat clear-bottom black plate at 4 × 10^4^ cells/well and allowed to attach for 24 h at 37 °C. Tests were performed after 24 h with 50 μM antioxidants (genistein and delphinidin) or three concentrations (50 and 100 μM 4-AT and NPs, and 150 μM 4-AT). After incubation, medium was removed and replaced with 10 µM H_2_DCF-DA or DHE (100 µL/well). A stock solution was prepared in DMSO, and a working solution was prepared in phosphate buffer. Fluorescence was measured at 490/529 nm (H_2_DCF-DA) and 475/579 nm (DHE) for 2 h at 37 °C (fluorescence measurement every minute). The sum of fluorescence values (“area under the curve”) was taken as a measure of ROS production.

### 4.9. The Content of Reduced and Oxidized Glutathione

The content of reduced and oxidized glutathione was estimated fluorimetrically with *o*-phtaladehyde (OPA) [66]. TAU and EP cells were seeded in 96-well clear plates at 4 × 10^4^ cells/well and allowed to attach for 24 h at 37 °C. Cells were treated with the tested antioxidants at a concentration of 50 μM (genistein, delphinidin) or 50, 100, or 150 μM (4-AT, NPs) for 24 h. After treatment, the medium was removed; the cells were washed with PBS (150 µL per well); and then 60 µL/well of cold lysis buffer containing 20 mM HCl, 5% TCA, 5 mM DTPA, and 10 mM L-ascorbic acid were added. The plate was shaken for 5 min and centrifuged for 5 min at 4000 rpm. Next, the cell lysate was transferred into two 96-well black bottom plates ((“+NEM”) and (“−NEM”)) at 25 µL/well. To the wells in the plate “+NEM”, 4 µL/well of freshly prepared 7.5 mM NEM in cold RQB buffer was added. Then, 40 µL/well of 1 M phosphate buffer (pH 7) was added to both plates, and the plates were shaken for 5 min at 700 rpm. Next, 160 µL/well of cold 0.1 M phosphate buffer (pH 6.8) and 25 µL/well of freshly prepared 0.5% OPA in methanol were added to both plates. Both plates were incubated for 30 min at room temperature with constant shaking. Fluorescence was measured at 355/430 nm. The concentration of reduced glutathione was determined by subtracting the fluorescence of the “−NEM” plate from the fluorescence of the “+NEM” plate and calculated with respect to the protein content. Protein content in cell lysates was determined by the method of Lowry et al. [67].

### 4.10. Estimation of Lipid Peroxidation with C11-BODIPY

TAU and EP cells were seeded in a 96-well flat clear-bottom black plate at a density of 4 × 10^4^ cells/well and allowed to attach for 24 h at 37 °C. Subsequently, cells were treated for 24, 48, and 72 h with genistein, delphinidin, 4-AT, and NPs at a concentration of 50 μM. Next, the medium was removed, 1 μM (final) fluorescent lipid peroxidation probe C-11 BODIPY was added (the stock solution was prepared in DMSO and diluted to a working solution in cell culture medium), and the cells were incubated for 30 min at 37 °C. From the fluorescence emission spectrum at the excitation wavelength of 460 nm, two wavelengths from the emission maximum were selected. Fluorescence was measured at 460/523 nm and 460/596 nm. The result was presented as the ratio of fluorescent intensity at 523 nm to 596 nm emission wavelengths.

### 4.11. Mitochondrial Mass Assessment

Cells were seeded at a density of 3 × 10^5^ cells/well into a 24-well plate and allowed to attach at 37 °C. Next, the cells were treated with genistein, delphinidin, 4-AT, and NPs at a concentration of 50 μM for 24 h. Subsequently, cells were trypsinized, counted, transferred to Eppendorf tubes, centrifuged at 1000 rpm for 5 min, washed with 1 mL of PBS, and centrifuged again. Next, 1 mL of 10 μM *N*-nonyl acridine orange (NAO) in PBS was added and the cells were incubated at 37 °C for 10 min. After incubation with NAO, the cells were centrifuged and washed with PBS (1 mL), and then the pellet was resuspended in 300 μL of PBS. Each sample was transferred into a 96-well black 96-well plate (100 μL/well; triplicates). Fluorescence was measured at 435/535 nm. The results were normalized to the cell number.

### 4.12. Evaluation of Changes of Mitochondrial Membrane Potential (ΔΨ_m_)

Changes in the mitochondrial membrane potential were evaluated using 5,5′,6,6′-tetrachloro-1,1′,3,3′-tetraethyl-imidacarbocyanine iodide (JC-1) with a Mitochondrial Membrane Potential Assay Kit. In mitochondria with high ΔΨ_m_, JC-1 forms complexes with profound red fluorescence, whereas in mitochondria that exhibit low ΔΨ_m_ levels, JC-1 persists as monomers and exhibits exclusively green fluorescence.

Briefly, TAU and EP cells were seeded in a 96-well flat clear-bottom black plate at a density of 4 × 10^4^ cells/well and allowed to attach for 24 h at 37 °C. The cells were treated with the studied compounds (50, 100, and 150 μM) for 24 h. Untreated cells were used as a control. Then, 10 μM (final) JC-1 staining solution was added to wells, and the plate was incubated at 37 °C for 30 min. After this time, the medium with JC-1 was gently removed and 100 μL of buffer included in the kit was added per well. Fluorescence was measured at 540/570 nm (red fluorescence) and 485/535 nm (green fluorescence). The results were presented as a green to red fluorescence intensity ratio.

### 4.13. Statistical Analysis

The results are presented as means ± SD from three independent experiments. To estimate the statistical significance of differences, ANOVA was used, except for simple comparisons between two groups where Student’s *t*-test was employed. *p* < 0.05 was considered as statistically significant and denoted as ^✠^ (Student’s test), * (ANOVA, differences with respect to untreated cells), or ^↓ ^(ANOVA, differences between various cells subjected to identical treatment). Statistical analysis of the data was performed using STATISTICA software package (version 13.1, Statsoft Inc. 2016, Tulsa, OK, USA).

## Figures and Tables

**Table 1:** Concentrations of compounds inducing 50% cytotoxicity (IC_50_) of TAU- and EP-transfected cells.

Compound	Cell Line	
	TAU	EP
H_2_O_2_	190 ± 7 μM ^✠^	312 ± 17 μM
6-OHDA	128 ± 4 μM	133 ± 9 μM
Genistein	100 ± 11 μM ^✠^	137 ± 12 μM
Delphinidin	141 ± 2 μM	133 ± 9 μM
4-Amino TEMPO	850 ± 157 μM	614 ± 26 μM
NPs	295 ± 37 μM	305 ± 4 μM

✠ *p* < 0.05 (TAU vs. EP).

## Data Availability

Not applicable.
